# Guidance for shared decision-making regarding orchiectomy in individuals with differences of sex development due to 17-β-hydroxysteroid dehydrogenase type 3 deficiency

**DOI:** 10.3389/fped.2025.1549400

**Published:** 2025-02-20

**Authors:** Lissa X. Yu, Jodie Johnson, Christine M. Pennesi, Michelle M. Ernst, Andrew Strine, Armand H. Matheny Antommaria, Robert J. Hopkin, David E. Sandberg, Behzad Khorashad, Lauren Mohnach, Amer Heider, Meilan M. Rutter

**Affiliations:** ^1^ Division of Pediatric and Adolescent Gynecology, Cincinnati Children’s Hospital Medical Center, Cincinnati, OH, United States; ^2^ Division of Human Genetics, Cincinnati Children’s Hospital Medical Center, Cincinnati, OH, United States; ^3^ University of Cincinnati College of Medicine, Cincinnati, OH, United States; ^4^ Division of Behavioral Medicine and Clinical Psychology, Cincinnati Children’s Hospital Medical Center, Cincinnati, OH, United States; ^5^ Division of Pediatric Urology, Cincinnati Children’s Hospital Medical Center, Cincinnati, OH, United States; ^6^ Ethics Center, Cincinnati Children’s Hospital Medical Center, Cincinnati, OH, United States; ^7^ Susan B. Meister Child Health Evaluation and Research Center, Department of Pediatrics, University of Michigan Medical School, Ann Arbor, MI, United States; ^8^ Department of Pediatrics, Division of Genetics, Metabolism and Genomic Medicine, C.S. Mott Children’s Hospital, Michigan Medicine, Ann Arbor, MI, United States; ^9^ Department of Pathology, Michigan Medicine, Ann Arbor, MI, United States; ^10^ Division of Endocrinology, Cincinnati Children’s Hospital Medical Center, Cincinnati, OH, United States

**Keywords:** DSD, decision-making, disorder of sex development, gonadectomy, intersex, orchiectomy, variation of sex development

## Abstract

17β-hydroxysteroid dehydrogenase type 3 deficiency is a 46,XY difference of sex development (DSD) that may present in childhood with inguinal testes or at puberty following virilization. We present four individuals, assigned female at birth, to highlight complexities and considerations surrounding orchiectomy. We reviewed the literature and created a “FACT sheet” to guide shared decision-making for patients, parents, and providers. “Ruth” presented at 16 months with inguinal herniae and underwent orchiectomy, based on parental preference. “Erica” presented at 13 years with voice deepening; she and her parents chose pubertal suppression and eventual orchiectomy. “Riley” presented at 18 months with inguinal herniae; after pubertal suppression and estrogen replacement, orchiectomy at age 13 years revealed germ cell neoplasia *in situ*. “Jordan” presented at birth with atypical genitalia and inguinal testes. Initially assigned female at birth, “Jordan's” sex was reassigned to male at 15 months; he subsequently underwent orchidopexy and expressed female gender identity at age 12 years. While early orchiectomy may eliminate pubertal androgens discordant with a female gender identity and remove malignancy risk, it necessitates pubertal induction and limits patient participation in decision-making. Fertility potential is unlikely; current fertility preservation protocols remain experimental for individuals with DSD. The stability of gender identity in this condition is difficult to predict. Postponing decision-making around testicular management allows the individual to meaningfully participate in the process and, if desired, undergo masculinizing puberty. Shared decision-making regarding testicular management requires consideration of predictions for gender identity stability, hormone replacement, testicular malignancy risk, and fertility potential.

## 1 Introduction

17β-Hydroxysteroid dehydrogenase type 3 (17βHSD3) deficiency is a 46,XY difference/disorder of sex development (DSD) (1). It is an autosomal recessive condition in which conversion of androstenedione to testosterone in testes is impaired (2, 3), resulting in the external genitalia of affected 46,XY newborns appearing female, although there are reports of atypical genitalia (4). Müllerian structures are absent, patients have a blind-ending vagina, and testes are located in the inguinal canals or labioscrotal folds. Because of the external genital appearance at birth, most children are raised as girls. At puberty, conversion of androstenedione to testosterone increases, resulting in virilization, with voice deepening, clitoral enlargement, hirsutism, and increased muscle mass.

From the perspective of clinical management, 17βHSD3 deficiency presents parents and healthcare providers with challenging decisions. The 2006 Chicago Consensus Statement (1) (hereafter referred to as the “consensus statement”) advised that decision-making regarding gender-of-rearing in DSD should encompass several factors, including diagnosis, genital appearance, surgical options, need for life-long hormone replacement, potential for fertility, views of the family, and circumstances relating to cultural practices. In cases of 17βHSD3 deficiency diagnosed in infancy, the consensus statement did not clearly recommend male gender assignment, instead urging that “the combination of a male gender identity in the majority and the potential for fertility (…unknown in 17βHSD3 deficiency) should be discussed when providing evidence for gender assignment” (1). In the international update of the DSD consensus statement, ten years later, the tone was more directive: “Male assignment is recommended for 17βHSD3 deficiency, since >50% later switch to male” (5). This guidance may be due to previous published studies from various countries where the majority of affected individuals are reportedly raised as girls from birth, but a spontaneous masculinizing puberty is associated with a change in gender identity (6, 7). Whether this change is attributable to prenatal and pubertal androgen effects on the brain, the experience of a contrasexual puberty, and/or reactions of the family and local cultural practices remains in dispute (8–11).

Complexities in clinical management of 17βHSD3 deficiency are reflected in a variety of decisions, including the choice of diagnostic methods, gender assignment, and associated decisions regarding the management of the testes (orchidopexy, orchiectomy), as well as the timing of procedures. These take into account expectations for stability of gender identity, risk of gonadal neoplasia, and efforts at fertility preservation (even if currently available only via research protocols). We present four cases to illustrate an admixture of all these decisions. The complexity of clinical decision-making, combined with the imperative of patient-centeredness as a value in high quality clinical care, has led us to create a Facts, Actions, Considerations, Time (FACT) sheet for gonad management. The FACT sheet is a synthesis of the relevant literature that can be practically applied in the clinical setting. The FACT sheet is offered as a path for clinicians engaging parents of young children with 17βHSD3 deficiency in the process of shared, proxy decision-making, or as a guide for older patients.

## 2 Illustrative cases

The four patients' clinical presentations illustrating decision-making surrounding testicular management are outlined below, with additional details summarized in the Supplementary Table S1. Pseudonyms are used to protect patient privacy. This report was deemed exempt by the Cincinnati Children's Institutional Review Board from ongoing review and was granted a waiver of informed consent due to patients having signed an institution-approved authorization form for personal health information disclosure for scientific presentation and publication purposes.

“Ruth”: Ruth, raised as a girl, presented at 16 months for inguinal hernia repair and was found to have bilateral inguinal testes and a 46,XY karyotype. She was presumed to have complete androgen insensitivity syndrome. Based on then standard practices, in the absence of commercially available genetic testing panels, she underwent androgen receptor sequencing that proved negative, followed by human chorionic gonadotropin stimulation testing that suggested and led to subsequent molecular confirmation of 17βHSD3 deficiency.

The interdisciplinary DSD team presented management options regarding the question of orchiectomy to her parents. The options of early orchiectomy, delaying orchiectomy to enable longitudinal gender assessment (with or without later pubertal suppression), and male gender re-assignment were discussed. Key parental concerns included gender identity stability and gonadal malignancy risk. Ruth's parents requested orchiectomy and continued to raise her as a girl. Over the years, her family met with her endocrinologist but declined more comprehensive psychosocial assessment or follow-up. Her family shared developmentally-appropriate information with her as she grew up, including inability to have biological children. At 12 years, Ruth expressed a strong wish and readiness to start estrogen. With guidance from their endocrinologist, Ruth's parents shared full information about her diagnosis and karyotype with her. The endocrinologist reviewed and validated this same information in detail in clinic and assessed her understanding of the effects of both estrogen and testosterone, before initiating hormone treatment. Ruth is now 16 years old, on transdermal estrogen, and requesting progressive dose escalation as appropriate for female puberty.

“Erica”: Erica, raised from birth as a girl, presented at age 13 years. She was a singer who presented to the otolaryngology voice clinic when her voice deepened from soprano to baritone and was subsequently referred to a DSD team. At her initial visit, she had terminal upper lip facial hair and clitoromegaly. Testing revealed a 46,XY karyotype and inguinal testes and gene sequencing confirmed 17βHSD3 deficiency.

Erica was enrolled in the DSD Translational Research Network, a registry of individuals with DSD that involves periodic, standardized assessments in the context of clinical care. She completed psychosocial screening (12) and met several times with the team psychologist. The Multifactor Gender Identity Scale (MGIS) (12) indicated that she was very content with being a girl, believed she behaved in a manner typical of girls, and felt some pressure to conform to gender stereotypes (but only in that she would not like it if she heard herself “talking like a boy”). The modified Body Image Scale (12) revealed overall positive body satisfaction, including with her “private parts.” Other screening measures did not identify any significant emotional or behavioral concerns. Erica, in her meetings with the team, reported a consistent female gender identity across time. She described an aversion to the masculinizing physical changes she was experiencing, including high distress about her voice deepening, particularly when it led to her misidentification as a boy (e.g., on the phone or in virtual meetings). Erica described a supportive family environment. The team identified no concerns related to Erica's ability to participate in shared decision-making, such as significant depression or altered mental status, or undue or negative pressure from family members.

After several shared decision-making conversations, Erica and her parents elected for pubertal suppression and, 8 months later, initiated estrogen replacement. Fourteen months post-diagnosis, with her family's support, Erica decided to proceed with orchiectomy with testicular cryopreservation under a research protocol to potentially preserve future fertility. Final pathology demonstrated immature testicular tissue and no malignant or pre-malignant cells. Germ cells were noted, although low in number.

“Riley”: Riley, assigned female at birth, was noted to have inguinal testes and an absent uterus at age 18 months when she presented for hernia repair; karyotype was confirmed as 46,XY. Riley's family was counseled about a likely diagnosis of complete androgen insensitivity syndrome and testes were left *in situ*. There was no further medical management or disclosure of a diagnosis until age 11 years, when Riley's parents sought out care from a DSD team after Riley developed signs of androgenization, including clitoromegaly, facial hair, and a deep voice. Targeted gene sequencing following suggestive biochemical testing confirmed the diagnosis of 17βHSD3 deficiency. Riley's completion of the MGIS, early in her care, revealed high gender contentedness along with relatively low gender typicality. This same pattern appeared in MGIS reports completed annually over the ensuing two years. Riley's happiness ratings regarding her body parts were “neutral” (neither happy nor unhappy), but she expressed unhappiness with her facial hair and excess body weight. Overall, behavioral and emotional functioning fell in the “normal” range, although both parents (using a standardized behavior problem checklist) reported Riley exhibiting a mild excess of depressive and anxious symptoms relative to population-based norms.

Based on clinical assessment and questionnaire data indicating a stable female gender identity, Riley and her parents agreed to pubertal suppression and starting estrogen. Riley's testes were monitored as she and her parents engaged in shared decision-making around orchiectomy including the use of a decision aid (13). A pelvic ultrasound at age 11 showed two sub-centimeter cysts in the right testis. With her parents' consent and Riley's assent, orchiectomy was performed at age 13. Pathology revealed bilateral dysgenetic testicles with an unexpected finding of microscopic germ cell neoplasia *in situ* in the left testis. Riley continues with transdermal estrogen replacement (7).

“Jordan”: Jordan, assigned female at birth, presented at age 13 months due to concerns for atypical genitalia. Diagnostic evaluation revealed inguinal testes, absent uterus, and a 46,XY karyotype. The presumed diagnosis was partial androgen insensitivity syndrome, but analysis of the androgen receptor gene was normal. Jordan's parents' perceptions that Jordan's behavior at that time suggested a “masculine personality” contributed to their decision to subsequently raise him as a boy. Following a robust response to topical testosterone, Jordan underwent orchidopexy and two-staged hypospadias repair. Also around this time, the parents were concerned Jordan had autism, and he was receiving intervention for “deficits in social communication.” At 23 months, Jordan was evaluated in a multidisciplinary developmental clinic and diagnosed with (“mild”) pervasive developmental disorder (not otherwise specified).

At age 8, whole exome sequencing became available and established the diagnosis of 17βHSD3 deficiency. At this age, Jordan's self-rating of gender typicality on the MGIS, as a boy, was very low, but with substantially higher gender contentedness. The family did not follow up with the DSD team; thus, no ongoing psychosocial or gender assessments were performed. At age 12, the family returned to clinic when Jordan began expressing a female gender identity and received treatment for depression, suicide ideation, and self-harming behavior. At age 13, Jordan was admitted to a psychiatric hospital for evaluation of self-injurious behavior. According to the parents, Jordan expressed the wish to be “pan-gender.” At age 14, Jordan progressed into puberty and was started on an androgen inhibitor, bicalutamide. While Jordan expressed interest in beginning estrogen therapy, the parents elected to delay further intervention towards gender reassignment until age 18.

## 3 Discussion

### 3.1 Gender and sex hormone considerations

Gender identity in individuals with 17βHSD3 deficiency is difficult to predict in infancy, as reports suggest changes in gender identity and gender dysphoria can occur during virilizing puberty in those raised as girls (2, 7). Our patients highlight decisions around orchiectomy influenced by gender of rearing at younger ages and gender identity in adolescence. A major consideration underpinning the decision for orchiectomy involves prevention of androgen production and virilization at puberty that are discordant in those raised as girls and who identify as female. Ruth was being raised as a girl when she presented in early childhood, whereas Erica and Riley expressed distress at experiencing androgen effects when they presented at puberty. Orchiectomy eliminates pubertal androgen production and necessitates pubertal induction and lifelong supplementation with estrogen (or testosterone, if desired). Conversely, retention of testes enables endogenous testosterone production (14), if desired in those who identify as male. Pubertal suppression can allow time for education and gender assessment, as was undertaken for Erica and Riley. An alternative temporizing approach, androgen inhibitor treatment, allows for testosterone production while preventing progression of androgen effects, as was undertaken for Jordan.

We searched the peer-reviewed literature to determine reported gender outcomes and possible influencing factors to guide shared decision-making around orchiectomy. This was a comprehensive search of all publications that included individuals who had a diagnosis of 17βHSD3 deficiency and documented long-term gender outcomes (gender outcomes from 12 years of age); orchiectomy status was ascertained, if reported. Our review yielded 35 relevant publications, which, with our four cases, included 120 individuals (40 originating from 17 families) (2, 4, 8, 10, 15–45). Of these, 114 were assigned female at birth and raised as girls (2, 4, 8, 10, 15–45). Pertinent to Ruth and other patients diagnosed by early childhood, we assessed individuals in whom early orchiectomy (before age 10 and onset of puberty) had been performed, and who had documented gender identity at/after puberty (after age 12). The extant literature suggested greater than 90% of those assigned female at birth who underwent early orchiectomy (12 individuals, including Ruth) maintained a female gender identity, but confidence regarding this outcome is limited by small numbers (Table 1a) (2, 4, 19, 38, 39). Related to this question, we also reviewed the association of pubertal status/age at diagnosis with long-term gender outcomes in the subset of 95 individuals for whom this information was ascertainable (2, 4, 8, 10, 15–45). This yielded similar findings (Table 1b), suggesting those who were raised female and diagnosed before virilizing puberty usually maintained female gender.

**Table 1a:** Long-term gender identity outcomes in individuals with 17**β**HSD3 deficiency initially raised female, with and without early orchiectomy.

Gender identity outcome^a^	Early orchiectomy^b^ (*N* = 12)	No early orchiectomy (*N* = 102)	All individuals (*N* = 114)
Female	11	71	82
Male	1	31	32
% Developing male gender identity	8%	30%	28%

^a^ Long-term gender identity outcome was defined as gender reported at age 12 years and older.

^b^ Early orchiectomy was defined as orchiectomy before age 10 years.

**Table 1b:** Long-term gender identity outcomes in individuals with 17**β**HSD3 deficiency initially raised female, grouped by pubertal status at diagnosis.

Gender identity outcome^a^	Diagnosis before puberty^b^ (*N* = 11)	Diagnosis at/after puberty^b^ (*N* = 84)	All individuals^c^ (*N* = 95)
Female	10	63	73
Male	1	21	22
% Developing male gender identity	9%	25%	23%

^a^ Long-term gender identity outcome was defined as gender reported at age 12 years and older.

^b^ Pubertal status at diagnosis was ascertained from documentation of when and how an individual presented (e.g., if diagnosed in infancy vs. if virilization with puberty had occurred).

^c^ Of the 114 individuals in Table 1a, the timing of diagnosis was unclear or unavailable for 19.

Erica and Riley were diagnosed after virilization at puberty. Both were determined by DSD teams to have female gender identities during adolescence, and orchiectomies were performed to support their preferences, following shared decision-making. Historical studies reported a 39%–64% female-to-male gender identity change post-puberty (7). However, more recent systematic reviews reported lower rates of 7%–10% female-to-male gender identity change (10, 24). Although these reviews indicate female-to-male gender identity change may be less frequent than originally reported, recent data included only probands, excluding some affected individuals within families. Our review of those raised female (not limited to probands) suggested a 28% female-to-male gender identity change overall (Table 1a), between past and recent estimates, and more specifically, 25% in those diagnosed at/after puberty and 9% in those who presented before puberty (Table 1b). We cannot conclude causality; future research (such as through collaborative national and international patient registries) should explore potential factors influencing long-term gender identity, including age of diagnosis, timing of orchiectomy, country/culture of origin, and patient phenotype.

In contrast, Jordan, who had atypical genitalia and was reassigned male and raised as a boy from age 13 months, developed virilization at puberty, yet expressed female gender identity at age 12 years. Complicating interpretation of the precipitants of Jordan's gender dysphoria are reports of the over-representation of autism spectrum disorder among adolescents experiencing gender dysphoria/gender nonconformity (46–48). In general, long-term gender outcome data on those raised male are sparse. There are a few reports of patients with atypical genitalia raised as boys from birth without information about gender outcomes beyond early childhood (10, 21, 49, 50), and two reported patients with atypical genitalia raised as boys with documented long-term male gender identity (10, 51). We did not identify any other cases with male-to-female gender identity change at/after puberty.

Other factors affecting gender outcomes in individuals with 17βHSD3 deficiency post puberty are unclear. A recent systematic review did not find an association between pubertal androgen levels and gender outcomes (10). A possible influence could be prenatal brain androgen exposure (which may be low in 17βHSD3 deficiency). There may be geographic, cultural or social influences: most European cases raised as girls identified as female post-puberty, while most reported cases with female-to-male gender identity change post-puberty were Asian, Latino, or African (10, 39). In families with multiple affected individuals, decisions regarding the proband's gender may have influenced decisions within the family. Of the 17 families with multiple affected individuals with long-term gender data, most (14/17) had consistent gender outcomes within families (2, 4, 10, 19, 20, 23, 26, 31, 35, 39, 40, 45). Of the nine families with individuals who experienced female-to-male gender change, most (7 out of 9) had another family member with the same change. There is no clear evidence for genotype-phenotype correlation with gender identity, even within families (4, 10, 23, 39, 45). Notably, the same molecular variant was associated with male gender identity in the Gaza Strip, but female gender identity in Brazil, suggesting sociocultural influences or genetic modifiers that vary with ancestry or ethnicity may be at play (23, 45).

In summary, children with 17βHSD3 deficiency raised as girls who present by early childhood, and who have undergone early orchiectomy to prevent pubertal androgenization, usually express female gender identity post-puberty, but data are limited. Some who present at puberty express female-to-male gender change, although recent literature suggests many maintain female gender identity and rates of change to male may be lower than previously reported. While consensus guidelines recommend strong consideration of male gender assignment in infants diagnosed with this condition (1, 5), our literature review and patient cases underscore the complexity of these decisions, as many individuals raised as girls maintain a female gender identity. It is important to support families through shared decision-making regarding gender-of-rearing and related decisions including testicular management.

### 3.2 Malignancy risk considerations

Germ cell malignancy risk is an oft-cited reason for gonadectomy in the DSD population. In the testes, precursor lesions to germ cell tumors are termed germ cell neoplasia *in situ* and can progress to germ cell tumors (52). It can be challenging to assess germ cell malignancy and premalignancy due to histological similarities in precursor lesions and immature testicular tissue (53). Due to the practice of early orchiectomy, there is little description of the natural history.

For individuals with 46,XY DSD, the risk of malignancy is highest in dysgenetic gonads and lowest in well-developed gonads. For those with 17βHSD3 deficiency, the consensus statement cited a moderate risk of 28% of premalignancy/malignancy; however, this was based on only 2 out of 7 patients with confirmed histopathology (1). A subsequent review cited a 5% risk, and recommended retention of testes being safe in patients with male gender identity, unless unable to be positioned in the scrotum (4). Our updated review of the literature included all publications that reported individuals with a diagnosis of 17βHSD3 deficiency in whom information on gonadal pathology or histopathology was available (Table 2). Including our cases, this yielded orchiectomy or testicular biopsy histopathologic descriptions of 39 patients with 17βHSD3 deficiency, of whom three had malignant or premalignant lesions (1, 4, 8, 10, 17, 18, 21, 24, 43, 53, 54). One was diagnosed with germ cell malignancy at age 30 and two with germ cell neoplasia *in situ* at ages 4 and 16 years (17, 18, 53). Of our patients, Riley had a premalignant lesion found at age 13. Therefore, including our case, the estimated premalignancy/malignancy risk appears to be 10% (4/39) (Table 2) with a 3% rate of malignancy based on published histopathology. However, given that gonads were historically removed following diagnosis and reports of pathological evaluation in adulthood are sparse, the natural history remains unknown, and we cannot estimate the frequency of malignancy if gonads were to remain *in situ*.

**Table 2:** Malignancies/pre-malignancies in individuals with 17βHSD3 deficiency with reported histopathology after orchiectomy.^f^

Author	Publication year	Total number of patients with documented histopathology	Age at orchiectomy (years)	Number of patients with malignancy/pre-malignancy	Age at malignancy/pre-malignancy diagnosis (years)	Gonad location	Malignant or pre-malignant histopathology^a^
Imperato-McGinley (18)^b^	1979	1	30	1	30	Inguinal	Teratocarcinoma-seminoma
Park (54)	1996	1	13	0			
Cools (53)^b^	2005	6	4^c^	1	4	Inguinal	GCNIS
Lee (21)	2007	11	0.5, 0.5, 1.5, 2, 2, 6, 12, 12, 14, 15, 20^d^	0			
Chuang (8)	2013	2^e^	2^e^, 28	0			
Galdiero (43)	2013	1	18^d^	0			
Mendonca (4)	2017	4	47, 53, 39, 33	0			
Yang (24)	2017	1	15	0			
Folsom (17)	2019	1	16	1	16	Inguinal	GCNIS
Krishnappa (10)	2022	4	16, 18, 20, 23^d^	0			
Wang (42)	2024	4^f^	2,2,10,11^f^				
Yu	Current series	3^e^	2^e^, 13, 15	1	13	Inguinal	GCNIS
Total		39		4			

Abbreviation: GCNIS, Germ cell neoplasia *in situ*.

^a^ GCNIS is used to encompass previous terminology for premalignant lesions, including carcinoma *in situ*, intratubular germ cell neoplasia (52).

^b^ References noted within 2006 Consensus Statement on Management of Intersex Disorders (1).

^c^ Patients ages 1 month to 23 years with multiple different diagnoses, ages of patients with 17βHSD3 not specified except one patient age 4 at orchiectomy (53).

^d^ These references note ages of evaluation and do not specify ages at orchiectomy (10, 21, 43).

^e^ Patient “Ruth” was previously reported in Chuang et al. and is therefore counted only once (8).

^f^ Specimens from Wang et al. were taken as testicular biopsies, in setting of orchiopexy rather than orchiectomy (42).

Cryptorchidism is an independent risk factor for testicular cancer, conferring up to 10% risk (55, 56). While surgical fixation of intra-abdominal or inguinal gonads into the labioscrotal folds may facilitate surveillance, this is not possible in all cases and there are no established guidelines or tumor markers for adequate surveillance. Surveillance methods include self-examination or ultrasound; sensitivity and specificity are unknown. The role of ultrasound, MRI, and gonadal biopsy for monitoring intra-abdominal testes in patients with DSD is unclear (57, 58).

### 3.3 Fertility potential considerations and preservation options

Individuals with 46,XY DSD are at risk of subfertility or infertility because of abnormal gonadal development, impaired gamete production, prior orchiectomy, and anatomic genitourinary development (59). There is a paucity of evidence on fertility potential of testes in DSD (60). One study reported the presence of germ cells as a possible indicator of fertility potential in 44 patients with various DSD diagnoses, following gonadectomy or gonadal biopsy (59). Germ cells were present in 68% of patients overall, varying by gonadal type (100% ovotestes, 81% ovaries, 75% testes, 73% dysgenetic testes and 15% streak gonads) and decreasing with age (88% patients between 0 and 3 years of age, 50% between 4 and 11, and 43% older than 12) (59).

Current evidence suggests that fertility potential for individuals with 17*β*HSD3 deficiency is low and spontaneous fertility is unlikely. Histologic studies have described arrested spermatogenesis with hyperplasia of Leydig and Sertoli cells and clusters of spermatogonia in young prepubertal patients, but immature or absent spermatogonia by puberty (61). There is a single self-reported account of fertility in an individual who was raised male and underwent genital reconstruction (62). It is unknown whether early orchidopexy would improve fertility potential of testes in this condition (60). In a paper published by Wang et al. (42), five individuals with 17βHSD3 deficiency were biopsied at the time of orchidopexy prior to commencing testosterone replacement therapy; biopsies showed various abnormal histopathologic findings including absence of lumens within spermatogenic tubules and absent Leydig cells. In addition, all but one lacked germ cells. The presence of scant germ cells, as seen in our patient “Erica”, does not conclusively suggest the potential for future fertility.

The 2016 updated consensus statement provided new emphasis on the importance of fertility due to expanding options for fertility preservation with advances in technology and changes in societal perceptions (5, 60). Of 1,040 adolescents and young adults with DSD surveyed, 55% of those unable to have biological children expressed a desire to have fertility treatments and 40% expressed an interest in trying new fertility techniques (63). Individuals with 46,XY DSD almost universally require assisted technologies to reproduce, such as testicular sperm extraction and *in vitro* fertilization. For those who undergo orchiectomy prior to puberty, testicular tissue cryopreservation is the only possibility for fertility preservation and remains experimental. This involves surgical removal of testicular tissue and cryopreservation for later use; its eventual success depends on the development of experimental techniques for maturation of spermatogonial stem cells into sperm (64). No successful retrieval of mature sperm or pregnancy has occurred to date from patients who underwent prepubertal testicular tissue cryopreservation. Research protocols are beginning to include patients with DSD; thus, future data may help inform the potential for fertility in this population (64).

Counseling around testicular tissue cryopreservation necessitates thorough discussion on its investigational nature, cost, ethical issues, possible need for preimplantation genetic testing, the genetic material being sperm (not oocytes), and concerns about safety of transplantation due to malignancy risk. It is also important to be attentive to cultural pressure regarding parenthood and fertility (65). We recommend that future histopathologic evaluation at time of orchiectomy include assessment of germ cell presence and maturation.

### 3.4 Surgical risk considerations

Surgical risks associated with orchiectomy include bleeding, infection, injury to adjacent organs, scarring, and need for additional surgery. Orchiectomy should be performed by a surgeon with expertise in this procedure. The permanency of orchiectomy, as it relates to endogenous hormone production and biologic fertility potential, should be reviewed in the shared decision-making process. The risks also include exposure to anesthesia, which may vary by patient age (although there is no conclusive evidence that a brief anesthetic in infancy results in neurodevelopmental sequelae) and the pediatric experience of the anesthesiologist (66).

### 3.5 Patient autonomy and decision-making considerations

Medical decision-making regarding orchiectomy for patients with 17βHSD3, especially those identified in infancy and childhood, is complex due to the heterogeneous potential benefits and risks and the uncertainties involved regarding gender dysphoria, malignancy, and fertility. For individuals presenting in infancy, like Ruth, Riley, and Jordan, the primary issues are gender assignment and testis management. Many are assigned female at birth based on external genital appearance. Gender assignment in those with atypical genitalia is more difficult because of limited information regarding gender identity in adulthood of those assigned male at birth.

The potential benefits of orchiectomy in infancy or early childhood include eliminating the risk of malignancy and preventing future pubertal hormones that are discordant with a female gender identity. The estimated malignancy/premalignancy risk may be about 10% and needs to be weighed against surgical risks. Orchiectomy might reduce the likelihood of gender dysphoria. In addition, if germ cells are present, there may be an opportunity for fertility preservation, although techniques are currently experimental. As gender identity is difficult to predict and can only be experienced by the individual, not performing orchiectomy in childhood allows patients to participate in decision-making and, if desired, undergo masculinizing puberty with endogenous hormones. Nevertheless, leaving the testes *in situ* raises the unanswered question of whether this might itself influence parents' gender socialization of their child and bias future gender development. A proposed alternative to pre-pubertal orchiectomy is the surgical fixation of intra-abdominal or inguinal gonads into the labioscrotal folds for surveillance. However, this is not always possible, and efficacy in terms of malignancy, gender identity, and fertility preservation is unclear.

For patients presenting in puberty, like Erica, or for those in whom decision-making regarding orchiectomy is deferred until the patient can participate, clinical choices include orchiectomy, time-limited pubertal suppression to permit more thorough evaluation of gender identity, and continued monitoring. If a patient is diagnosed during puberty, it is essential to educate the patient about their diagnosis and include the patient in medical decision-making. Decision-making may be strongly influenced by the patient's gender identity and its stability. If the patient unequivocally expresses a male gender identity, the primary issue is weighing the risk of malignant transformation against the need for hormone replacement. If the patient firmly identifies as female, there are substantially fewer reasons to retain the testes; hormone replacement will be required, and orchiectomy will eliminate risk of malignant transformation and need for pubertal suppression. Fertility preservation remains a secondary consideration due to the methods currently available to this patient population being experimental.

### 3.6 Informed and shared decision-making

Decisions around orchiectomy for individuals with 17βHSD3 deficiency are complex. There is no single treatment plan in which the potential benefits substantially outweigh the risks, particularly for patients presenting in infancy. Shared decision-making is essential as individual patients and family members place different values on each outcome (67, 68). Informed consent (and assent) for treatment is best conducted by a multidisciplinary team who can describe these potential risks and the uncertainties surrounding them, and answer questions about them. Given the complexities involved, we created a 17βHSD3 deficiency condition-specific FACT sheet to guide education and shared decision-making between patients, families, and primary care and specialist providers in the delivery of patient- and family-centered clinical care (Table 3). While this particular DSD condition is rare, the considerations presented extend to other DSD, albeit with condition-specific nuances, and provide valuable guidance on shared decision-making for health providers who care for patients with DSD.

**Table 3:** FACT sheet: considerations for choosing or not choosing surgery to remove the testicles for people with 17-beta-hydroxysteroid dehydrogenase type 3 (17βHSD3) deficiency.

**Facts:** Things to know about 17βHSD3 deficiency before deciding.		
Hormones and gender identity	Most children with 17βHSD3 deficiency are raised as girls because of the appearance of their genitals at birth. Some are raised as boys when the genitals are neither clearly male nor female. At puberty, some individuals with 17βHSD3 deficiency may change their gender from female to male, while others continue to identify as girls and women. It is difficult to predict which children with 17βHSD3 deficiency will grow up to identify as which gender(s). We do not know why some remain in the gender in which they were raised, and others do not. We do know that removing the testicles before puberty prevents masculine body changes at puberty. Doing so does not guarantee that an individual will continue to identify with the gender in which the individual was raised. Treatment to “pause” or block puberty for a limited time is an option. This treatment can allow more time for careful decision-making.	
Cancer risk	If left in the body, there is a risk for cancer of the testicles. Based on the small amount of information available, we estimate 10 out of 100 people with 17βHSD3 deficiency will develop pre- or early cancer changes in the testicles and 3 out of 100 will develop actual cancer. It may be safe to leave the testicles in place and check them regularly. However, we do not have enough information to know the true risk of leaving the testicles in place long term, and methods of checking are not perfect.	
Fertility	Fertility is the ability to have biological children. We are not sure if people with 17βHSD3 deficiency are able to have children. The possibility may get better with advances in science. If the testicles are removed, they can be frozen. We hope in the future to develop ways to thaw them and collect sperm.	
**Actions (Options):** What are the choices?		
Options	Surgery—**remove** the testicles	No surgery—**keep** the testicles
**Considerations:** Making a Choice—What is most important to you?		
Consider	Reasons **for removing** the testicles	Reasons **for keeping** the testicles
Hormones	• Prevent hormones at puberty that cause male changes and disagree with female gender identity • Hormone treatment that agrees with a female gender identity can be used at puberty • Takes away the need for medicine to block hormones at puberty	• Have natural hormones at puberty that agree with a male gender identity • May not need long-term hormone treatment from puberty onward • Medical treatment to block puberty can allow time to decide
Gender	• Removing the testicles at a young age in an individual being raised as a girl might contribute to her identifying as a woman when older • Many girls will continue to identify as girls or women after puberty starts	• Future gender identity is difficult to predict • Some girls identify as boys or men after puberty starts
Cancer risk	• Remove any risk or worry for cancer • The ways to check testicles for pre-cancer changes are not perfect	• Cancer risk is unknown and may be low • There are ways to check testicles for pre-cancer changes
Fertility	• The possibility for fertility is uncertain and likely low • The testicles can be frozen. There is research to collect sperm from frozen testicles	• Surgery permanently prevents natural fertility • The research on collecting sperm from frozen testicles may not be successful
Surgery risks	• The risks and complications are low with experienced surgeons and anesthesiologists	• There are risks and complications from surgery and anesthesia
Who decides?	• Parents make the decision for their child if the child is young. This might prevent difficult and stressful decisions when the child is older	• Not doing surgery when the child is young allows the child to participate in the decision when the child is older
**Time:** Take time to decide. Learn about 17βHSD3 deficiency and the choices. Surgery to remove the testicles is not urgent.		
