# Supplementary material for: Guidance for shared decision-making regarding orchiectomy in individuals with differences of sex development due to 17-β-hydroxysteroid dehydrogenase type 3 deficiency
Source: Front Pediatr. 2025 Feb 20;13:1549400. doi: 10.3389/fped.2025.1549400 (PMC11882584; doi:10.3389/fped.2025.1549400)
Supplement: Supplementary file 1 [file Table1.docx]

Table 1. Clinical details of patients with 46,XY DSD due to 17βHSD3 deficiency

|  | **“Ruth”^*^: Presentation in childhood** | **“Erica”: Presentation in adolescence** | **“Riley”: Gonadal tumor** | **“Jordan”: Gender dysphoria** |
| --- | --- | --- | --- | --- |
| **Gender** | Assigned female at birth, currently identifies as female. | Assigned female at birth, currently identifies as female. | Assigned female at birth, currently identifies as female. | Assigned female at birth. Reassigned male by parents at age 15 months. Began identifying as female at age 12 years. Expressed wish to be “pan-gender” at age 13 years. |
| **Presentation** | 16 months during inguinal hernia repair. | 13 years with voice deepening from soprano to baritone. | 18 months during inguinal hernia repair - presumed to be CAIS. Presented again at age 11 years with masculinization. | Infancy with atypical genitalia. |
| **Physical examination** | 16 months: Bilateral inguinal herniae, typical female genitalia. | 13 years: Deep voice, hirsutism, Tanner IV pubic hair, clitorophallic stretched length 5.0 cm and width 1.3 cm, normal labia majora and minora without posterior labial fusion, non-palpable gonads, blind-ending vagina measuring 2 cm. | 18 months: Bilateral inguinal herniae, typical female genitalia.  11 years: Enlarged clitoris, rugated labia majora, Tanner III breast development, voice cracking, tall stature. | 13 months: Perineal urogenital sinus opening, clitorophallic stretched length 2.0 cm, slight rugation of labio-scrotal folds, palpable inguinal gonads. |
| **Internal anatomy** | Intraoperative assessment (16 months): Bilateral inguinal testes (confirmed on biopsy), vas deferens, spermatic vessels entering internal inguinal ring, blind-ending vagina, absent Mullerian structures. | Ultrasound (13 years): Bilateral inguinal testes, 2-3 microcalcifications on right, absent Mullerian structures.  Intraoperative assessment (15 years): Bilateral inguinal testes, vas deferens, underdeveloped epididymides, absent Mullerian structures. | Intraoperative assessment (18 months): Bilateral inguinal testes (not confirmed with biopsy), epididymides, vas deferens, blind-ending vagina measuring 4 cm, absent Mullerian structures.  Ultrasound (11 years): Bilateral inguinal testes, 2 subcentimeter cysts on right, vagina present, absent Mullerian structures. | Intraoperative assessment (15 months): Bilateral inguinal testes, blind-ending vagina measuring 1.5 cm, absent Mullerian structures. |
| **Hormonal testing** | Low testosterone to androstenedione ratio (0.59) post-hCG stimulation. | Low testosterone to androstenedione ratio (0.36). At age 13 years, testosterone in lower pubertal range (226 ng/dL). | Low testosterone to androstenedione ratio (0.34). | Low testosterone to androstenedione ratio (0.22) post-hCG stimulation. At age 14 years, testosterone in lower pubertal range (130 ng/dL). |
| **Gene sequencing** | *HSD17B3* gene sequencing with two variants:  - Heterozygous: c.238C>T (p.Arg80Trp), missense variation, pathogenic  - Heterozygous: c.277+4A>T, splice donor site variation, pathogenic | *HSD17B3* gene sequencing with two variants:  - Heterozygous: c.845C>T (p.Pro282Leu), missense variation, pathogenic  - Heterozygous: c.391A>T (p.Asn131Tyr), missense variation, uncertain clinical significance | *HSD17B3* gene sequencing with two variants:  - Heterozygous: c.277+4A>T, splice donor site variation, pathogenic  - Heterozygous: c.352G>T (p.Glu118*), missense variation, likely pathogenic | Whole exome sequencing with two variants in the *HSD17B3* gene:  - Heterozygous: c.277+4A>T, splice donor site variation, pathogenic  - Heterozygous: c.278-1G>C, splice acceptor site variation, pathogenic |
| **Testis management and pathology results** | Orchiectomy at age 2 years.  Bilateral immature testes with no pathologic abnormality, non-proliferating primitive germ cells in a normal distribution within tubules, no germ cell neoplasia. | Orchiectomy at age 15 years.  Bilateral immature testes, no malignancy. Germ cells present, low in number. | Orchiectomy at age 13 years.  Left dysgenetic testicle with microscopic germ cell neoplasia *in situ* and calcifications, OCT4 immunostain confirmatory; right dysgenetic testicle with calcifications, no germ cell neoplasia *in situ* seen by OCT4 immunostain; SALL4 marked scattered germ cells. | Orchiopexy at age 15 months. |
| **Hormone therapy** | Pubertal induction with transdermal estrogen initiated at age 12 years, remains on this treatment at age 16 years. | Puberty suppressed with leuprolide at age 13 years for 6 months, followed by histrelin subdermal implant until orchiectomy at age 15 years. Transdermal estrogen initiated at age 14 years, remains on this treatment at age 17 years. | Received histrelin subdermal implant and started transdermal estrogen at age 11 years. Orchiectomy at age 13 years, remains on transdermal estrogen at age 16 years. | Started anti-androgen bicalutamide at age 14 years, remains on this treatment at age 17 years. |

Abbreviations: 17βHSD3, 17-beta-hydroxysteroid dehydrogenase-type 3; CAIS, complete androgen insensitivity syndrome; hCG, human chorionic gonadotropin

^*^Names are pseudonyms.
